# Immunogenic and efficacious SARS-CoV-2 vaccine based on resistin-trimerized spike antigen SmT1 and SLA archaeosome adjuvant

**DOI:** 10.1038/s41598-021-01363-7

**Published:** 2021-11-08

**Authors:** Bassel Akache, Tyler M. Renner, Anh Tran, Lise Deschatelets, Renu Dudani, Blair A. Harrison, Diana Duque, Julie Haukenfrers, Martin A. Rossotti, Francis Gaudreault, Usha D. Hemraz, Edmond Lam, Sophie Régnier, Wangxue Chen, Christian Gervais, Matthew Stuible, Lakshmi Krishnan, Yves Durocher, Michael J. McCluskie

**Affiliations:** 1 National Research Council Canada, Human Health Therapeutics, 1200 Montreal Road, Ottawa, ON K1A 0R6 Canada; 2 National Research Council Canada, Aquatic and Crop Resource Development, 6100 Avenue Royalmount, Montreal, QC H4P 2R2 Canada

**Keywords:** Adjuvants, Protein vaccines, Infectious diseases, Vaccines

## Abstract

The huge worldwide demand for vaccines targeting SARS-CoV-2 has necessitated the continued development of novel improved formulations capable of reducing the burden of the COVID-19 pandemic. Herein, we evaluated novel protein subunit vaccine formulations containing a resistin-trimerized spike antigen, SmT1. When combined with sulfated lactosyl archaeol (SLA) archaeosome adjuvant, formulations induced robust antigen-specific humoral and cellular immune responses in mice. Antibodies had strong neutralizing activity, preventing viral spike binding and viral infection. In addition, the formulations were highly efficacious in a hamster challenge model reducing viral load and body weight loss even after a single vaccination. The antigen-specific antibodies generated by our vaccine formulations had stronger neutralizing activity than human convalescent plasma, neutralizing the spike proteins of the B.1.1.7 and B.1.351 variants of concern. As such, our SmT1 antigen along with SLA archaeosome adjuvant comprise a promising platform for the development of efficacious protein subunit vaccine formulations for SARS-CoV-2.

## Introduction

Efforts to combat the COVID-19 pandemic have focused largely on the generation of novel vaccines against its etiological agent, Severe Acute Respiratory Syndrome Coronavirus 2 (SARS-CoV-2). Based on existing data from other coronaviruses, namely the original SARS-CoV, SARS-CoV-2 spike glycoprotein has been chosen as the target antigen for multiple vaccine strategies due to its importance in mediating cellular entry, accessibility on the viral surface and its potentially strong immunogenicity^1^. Antibodies capable of disrupting the interaction between the receptor-binding domain (RBD) of the spike glycoprotein and the cellular ACE2 receptor reduce infection and mediate protection^2^.

A number of vaccine platforms have been utilized against SARS-CoV-2, including novel approaches based on mRNA/lipid nanoparticles or viral vectors. Speed of development/fabrication was a key advantage with these genetic vaccines where the spike protein is simply encoded instead of produced, allowing them to rapidly enter clinical trials. Based on their high efficacy in preventing infection, a number of vaccines, namely those by Pfizer/BioNtech, Moderna, Janssen and AstraZeneca/University of Oxford were granted emergency approval in multiple countries worldwide^3–9^. However, while highly efficacious, these types of vaccines do have some drawbacks, such as the high cost of mRNA vaccine formulations, as well as their relatively poor stability, requiring more stringent long-term storage temperatures^10^. In addition, long term effects of repeated administration of mRNA vaccines is not known. As for the adenoviral-based vaccines, they are associated with manufacturability challenges^11^ and the induction of anti-vector antibodies that could potentially reduce the efficacy of future immunizations with a similarly pseudotyped vector^12,13^. Furthermore, the mRNA and adenoviral-based vaccines have been linked to rare cases of severe anaphylaxis and potentially fatal thrombotic thrombocytopenia, respectively^14–16^.

Protein subunit vaccines have a long track record of efficacy and safety against multiple infectious diseases, such as Hepatitis B, Meningitis B and Shingles^17,18^. They are also proven to be safe in pediatric and vulnerable populations. Importantly, they are not associated with the main disadvantages of RNA/viral vaccines mentioned above. Novavax’s protein subunit vaccine adjuvanted with Matrix-M™ has shown strong efficacy in clinical trials so far^19,20^ with no serious adverse events attributed to the vaccine. Other subunit vaccine formulations including adjuvants, such as AS03 or the TLR9 agonist CpG, are efficacious in preclinical models^21^. However, in face of extremely high global demand, there is a need for novel vaccine formulations based on new antigens and/or adjuvants that are ideally low cost and can be manufactured/shipped easily. Also, with the emergence of viral variants, highly immunogenic vaccines capable of inducing strong humoral and cellular immune responses would be more likely to cross-protect against so-called SARS-CoV-2 variants of concerns, such as B.1.1.7 and B.1.351, originally identified in UK and South Africa, respectively. While antigen-specific T cell responses cross-react well to the SARS-CoV-2 variants, mutations in B.1.351 spike protein have been shown to make it more resistant to antibodies developed against the spike protein of the reference strain, originally identified in Wuhan^22–24^.

Ideally, a vaccine antigen would not only be immunogenic but could be quickly produced with high yields. This would enable quicker delivery and reduced costs; both important factors when trying to respond to a pandemic on a global scale. We have developed a method to generate full-length SARS-CoV-2 spike ectodomain protein in our proprietary Chinese Hamster Ovary clone (CHO2353™), which is based on cumate-inducible expression technology^25^ modified to permit higher level protein expression. To more closely mimic the conformation of the native spike glycoprotein, which exists on the viral surface as a trimer, a trimerization partner was fused to the C-terminus of the spike ectodomain. T4 phage foldon domains have been employed previously for this purpose when generating spike antigens^26–28^. However, as a non-human protein, it is likely to induce immune responses to itself in addition to the desired viral spike domains. Utilizing an alternative approach, we fused human resistin, a naturally trimeric hormone, to our spike ectodomain to successfully generate stable trimeric spike glycoprotein: SmT1. Due to the phenomenon of immunological self-tolerance^29^, utilization of our resistin fusion protein in a vaccine formulation should focus immune responses mainly on the SARS-CoV-2 domains. We were able to produce SmT1 antigen at very high purity and yield post-purification (~ 370 mg/L)^30,31^. Our approach also yields a more highly homogenous trimeric preparation than with spike fused to the T4 foldon or GCN4 trimerization domains^30^. Based on previous reports, we sought to further improve the stability and purity of our spike protein by abolishing the S1/S2 cleavage site and introduced stabilizing prolines^27,32^. Plasma samples from human SARS-CoV-2 convalescent patients strongly bound our human resistin-fused spike glycoprotein in a plate-based immune assay, indicating that its conformation is similar to that encountered by our immune system upon infection^31,33^.

Due to an unprecedented demand for vaccines, adjuvants are in short supply, highlighting the need to develop new efficacious formulations. We have developed a liposomal adjuvant based on archaeal glycolipids, sulfated lactosyl archaeol (SLA) archaeosomes^34^. SLA (6′-sulfate-β-d-Galp-(1,4)-β-d-Glcp-(1,1)-archaeol) is a glycolipid consisting of a sulfated lactose polar head group fused to archaeol. Archaeol has unique adaptations not found in eukaryotic or bacterial lipids, namely (1) an ether linkage between the glycerol backbone and the lipid tails and (2) lipid tails named phytanyl chains composed of repeating branched 5-carbon repeating units^35^. In previous preclinical studies, we have demonstrated the safety of liposomes formed with SLA and their ability to induce strong humoral and cellular immune responses to multiple antigens^36–39^. Herein, we evaluated the immunogenicity of our novel spike protein in mice and hamster models either alone or in combination with adjuvants including SLA archaeosomes, aluminum salts and TLR agonists. Vaccine formulations were shown to be strongly immunogenic, inducing robust antigen-specific antibody and T cell responses which were dependent on adjuvant tested. Vaccine formulations were highly efficacious in a hamster challenge model, with a single dose mediating protective immunity. Furthermore, the immunogenicity was sufficient to mediate cross-neutralization activity against the spike protein of multiple variants. Thus, we present a novel strongly immunogenic and efficacious vaccine formulation that could help address the global crisis caused by SARS-CoV-2 as it continues to evolve and spread worldwide.

## Results

### Antibody response to SmT1 antigen

**Figure 1:**
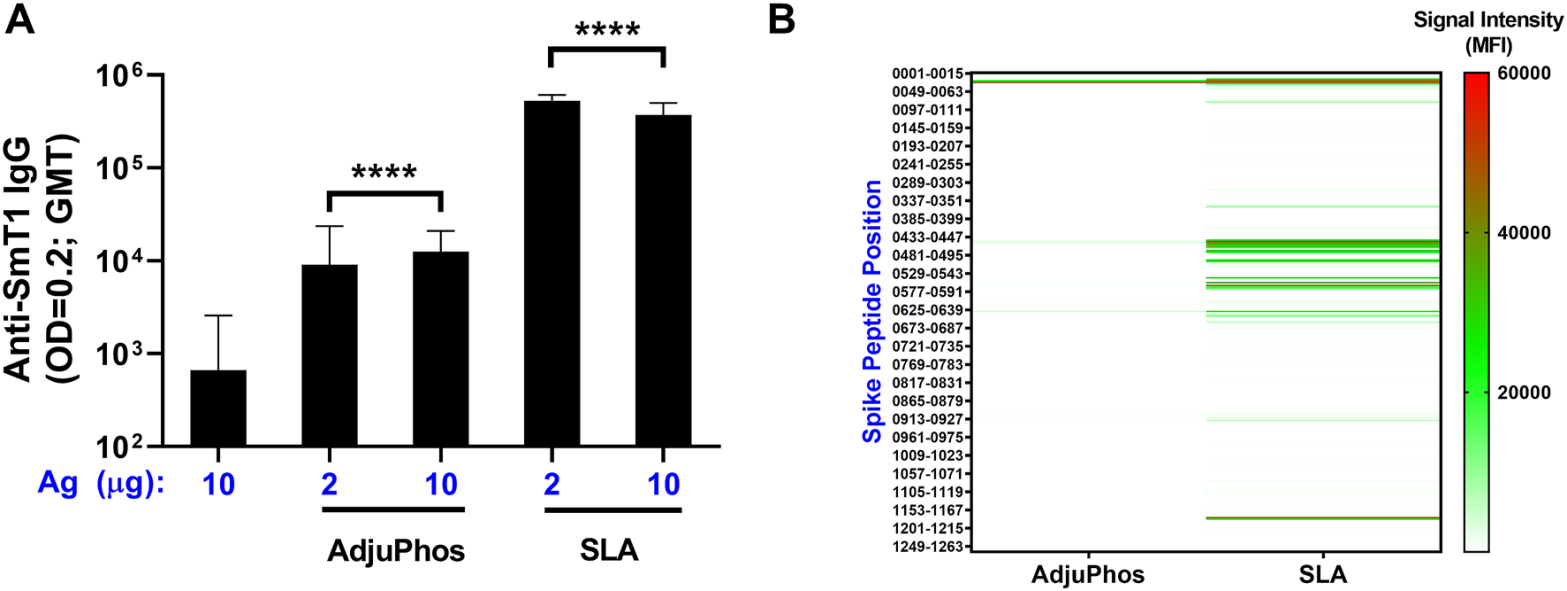
Levels and specificity of antibodies induced by SmT1-based vaccine formulations. C57BL/6 mice (n = 8/group) were immunized i.m. with SmT1 (2 or 10 µg) with or without adjuvant on Days 0 and 21. Serum was collected on Day 28 and analyzed by ELISA to determine the antibody titers (**A**). Grouped data is presented as geometric mean + 95% confidence interval. Statistical significance of differences for the adjuvanted groups vs. the antigen alone control group is shown: ****p < 0.0001 by one-way ANOVA followed by Tukey's multiple comparisons test. Serum (n = 5–6 per group) was also analyzed by peptide microarray to identify specific epitopes recognized by the vaccine-induced IgG antibodies (**B**). The median of the mean fluorescent intensity values obtained with each 15mer peptide are represented.

Mice (n = 8 per group) were immunized on Days 0 and 21 with SmT1 antigen alone or adjuvanted with the aluminum phosphate-based adjuvant, AdjuPhos, or SLA archaeosomes. For the adjuvanted formulations, two different antigen doses (2 or 10 µg) were tested, while 10 µg of antigen was used for the antigen alone control group. The latter induced geometric mean titers (GMT) of 667, whereas immunization with 2 µg of SmT1 + Adjuphos resulted in significantly higher titers of 9,051 (Fig. 1A, p < 0.0001). Antibody titers in animals immunized with 2 µg of SmT1 + SLA (GMT of 524,812) were significantly higher than those obtained with antigen alone or AdjuPhos-adjuvanted formulations (p < 0.0001). When delivered with the same adjuvant, similar antibody titers were generated whether 2 or 10 µg of antigen was administered. The specificity of the polyclonal antibody repertoire induced by our vaccine formulations was evaluated using a short peptide array spanning the entire length of the SARS-CoV-2 spike protein. Serum from 5–6 animals immunized with 2 µg of antigen adjuvanted with SLA archaeosomes or AdjuPhos was tested separately on the peptide array. The IgG antibodies induced by the SLA-adjuvanted formulation generated strong binding to various hotspots located across the length of the protein (Fig. 1B), with a mean fluorescence intensity greater than 15,000 seen in peptides stretching amino acids 17–43 in the N-terminal domain (NTD), 441–487 & 493–511 in the RBD, 541–583 & 629–643 in S1 and 1173–1191 in S2. While samples from the AdjuPhos/spike-vaccinated mice resulted in weaker signals (likely due to the lower antibody titers), binding was generally observed in the same amino acid residues as with SLA, with the strongest binding seen in the NTD. As we did detect antibodies targeting the critical RBD, we were encouraged to further evaluate the SLA-adjuvanted vaccine formulation.

### Comparison of antigen formats

**Figure 2:**
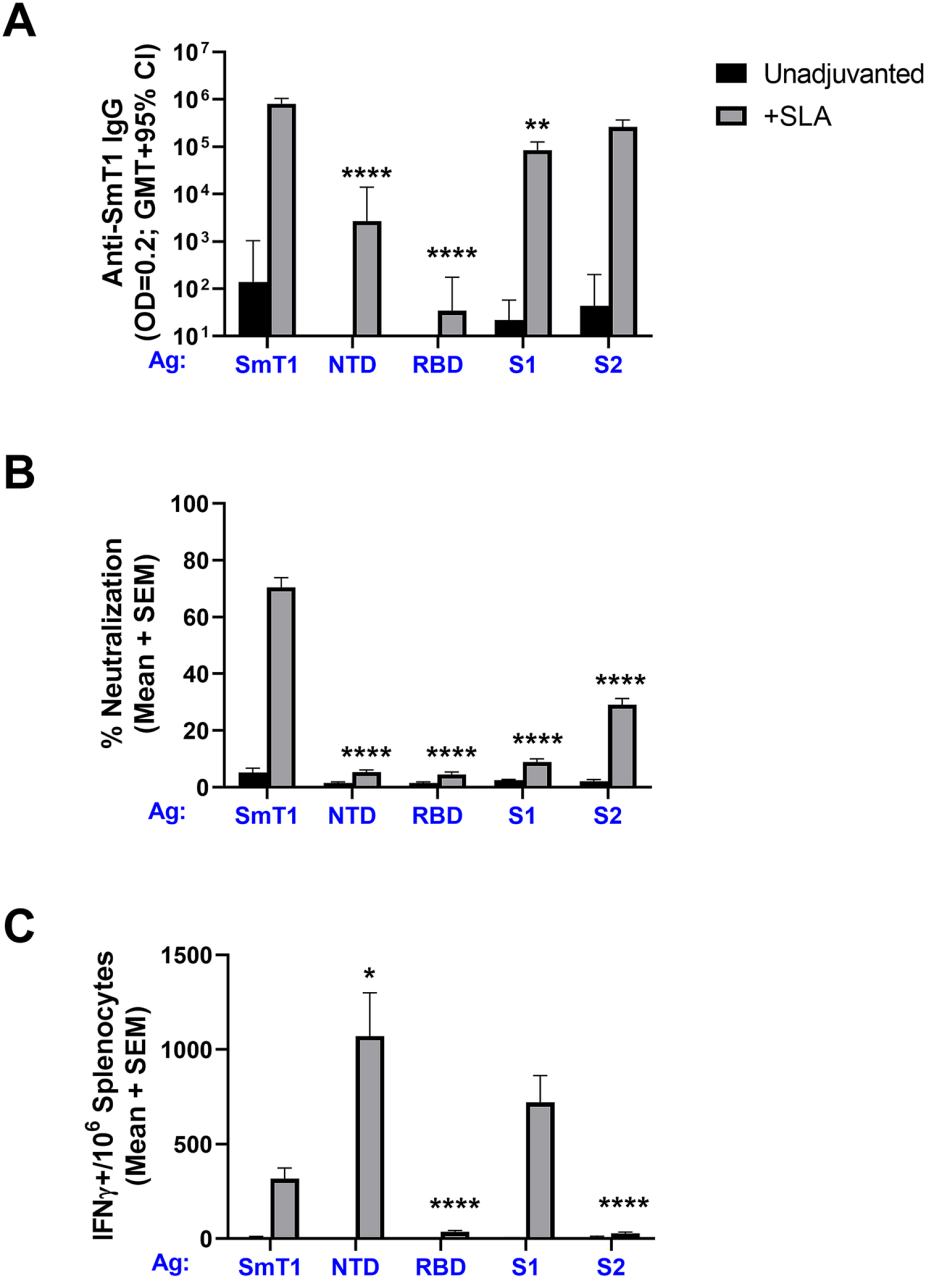
Comparison of immunogenicity of various spike-derived antigen formats delivered with SLA adjuvant. C57BL/6 mice (n = 10/group) were immunized i.m. with 2 µg of SmT1, spike NTD, RBD, S1 or S2 alone or with SLA archaeosomes on Days 0 and 21. Serum was collected on Days 28 and analyzed by ELISA to determine the antibody titers (**A**). Grouped data is presented as geometric mean + 95% confidence interval. Serum from Day 28 was also analyzed at a final serum dilution of 1:75 by surrogate neutralization assay (**B**). Grouped data is presented as mean + standard error of mean. Splenocytes were harvested on Day 28 and analyzed by IFN-γ ELISpot when stimulated by spike peptide pools (**C**). Values obtained with media alone were subtracted from those measured in the presence of the peptides. Grouped data is presented as mean + standard error of mean (SEM). Statistical significance of differences for the adjuvanted subdomain groups vs. SmT1 + SLA group is shown: *p < 0.05, **p < 0.01 and ****p < 0.0001 by one-way ANOVA followed by Tukey's multiple comparisons test.

As vaccine efficacy has been tied to the induction of immune responses to specific domains of the spike protein, we sought to compare the immunogenicity of our full-length SmT1 to subdomains consisting of NTD (a.a. 16–307), RBD (a.a. 331–521), S1 (a.a. 16–686) and S2 (a.a. 687–1208). Mice (n = 10 per group) were immunized on Days 0 and 21 with 2 µg of the various antigens alone or adjuvanted with SLA. Following a second vaccine dose, most of the antigen formats (when adjuvanted with SLA) were shown to induce high levels of antigen-specific antibodies as determined by ELISA, while weak responses were induced by antigen alone (Fig. 2A). The smallest antigen, RBD, induced low levels of Ab titers: GMT of 34, while the SmT1 + SLA induced the highest levels of antibody titers (814,117). The levels with SmT1 were significantly higher than those obtained with NTD, RBD or S1 + SLA (p < 0.01). Titers with S2 + SLA were ~ threefold lower than with SmT1, but this difference did not reach a level of statistical significance. To investigate the functionality of these antibodies, a surrogate cell-based neutralization assay was employed. Immunized mouse serum was able to inhibit the binding of biotin-labeled spike to the surface of Vero E6 cells that naturally express ACE2 receptor. The SmT1 + SLA induced significantly higher neutralization than all other groups (p < 0.0001), with an average of 71% neutralization (Fig. 2B). The sera of mice immunized with S2 + SLA also had measurable neutralizing activity (29%), which was significantly higher (p < 0.0001) than obtained with sera from mice immunized with SLA-adjuvanted NTD, RBD or S1. Finally, as cellular immune responses may also play a role in controlling viral infections, the levels of antigen-specific T cells were measured in these mice by IFN-γ ELISpot. Antigen-specific T cells were induced by a number of antigen formats when adjuvanted with SLA archaeosomes (Fig. 2C). The three antigen formats containing the NTD induced the highest levels of IFN-γ^+^ SFCs. NTD, S1 and SmT1 induced an average of 1071, 721 and 316 IFN-γ^+^ SFCs/10^6^ splenocytes, respectively, with the levels induced by NTD being significantly higher than those obtained with SmT1 (p < 0.05). SmT1 was selected for further testing due to its superior neutralization data and as a larger protein, it would yield a greater number of potential T cell epitopes in a heterogeneous human population.

### Antigen dose response to SmT1 antigen

**Figure 3:**
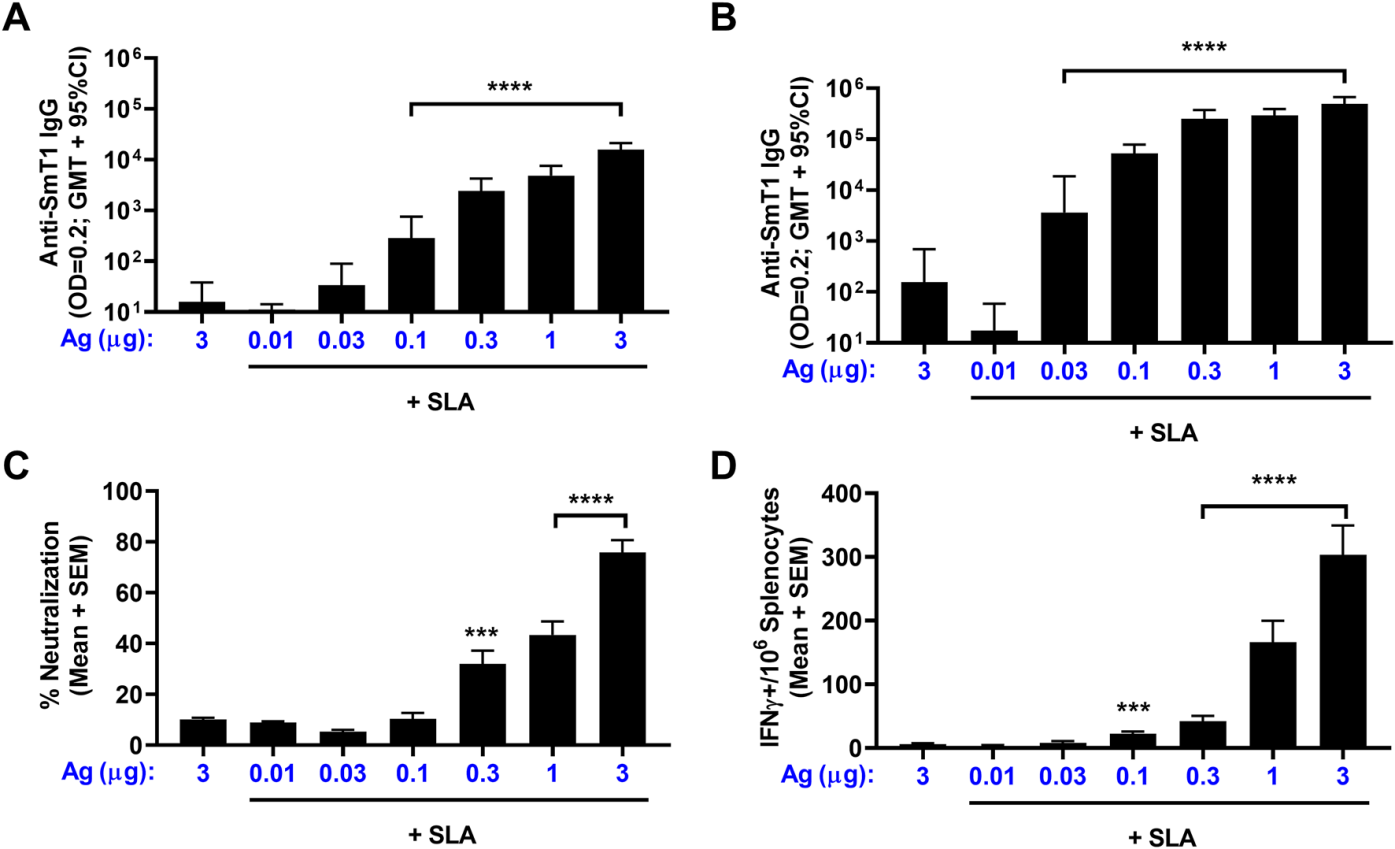
Antigen dose response of SmT1-SLA adjuvanted vaccine formulations. C57BL/6 mice (n = 10/group) were immunized i.m. with 3 µg of SmT1 alone or 0.01–3 µg of antigen with SLA archaeosomes on Days 0 and 21. Serum was collected on Days 20 (**A**) and 28 (**B**) and analyzed by ELISA to determine the antibody titers. Grouped data is presented as geometric mean + 95% confidence interval. Serum from Day 28 was also analyzed at a final serum dilution of 1:75 by surrogate neutralization assay (**C**). Grouped data is presented as mean + standard error of mean. Splenocytes were harvested on Day 28 and analyzed by IFN-γ ELISpot when stimulated by spike peptide pools (**D**). Values obtained with media alone were subtracted from those measured in the presence of the peptides. Grouped data is presented as mean + standard error of mean (SEM). Statistical significance of differences for the adjuvanted groups vs. the antigen alone control group is shown: ***p < 0.001 and ****p < 0.0001 by one-way ANOVA followed by Tukey's multiple comparisons test.

To better understand the immunogenicity of our SmT1-SLA adjuvant vaccine formulation, an antigen dose response study was conducted, where mice (n = 10 per group) were immunized on Days 0 and 21 with 3 µg SmT1 antigen alone or 0.01 to 3 µg of antigen adjuvanted with SLA archaeosomes. After a single vaccine dose, animals immunized with SLA-adjuvanted formulations containing as little as 0.1 µg of antigen had significantly higher Ab titers than those receiving unadjuvanted antigen (Fig. 3A; p < 0.0001). Following a second vaccine dose, formulations with as little as 0.03 µg Ag adjuvanted with SLA induced significantly higher titers than 3 µg Ag alone: GMT of 3,633 vs. 157 (Fig. 3B; p < 0.0001). While 3 µg of SmT1 + SLA induced the highest levels of antibody titers (494,160), they were not significantly higher than those obtained with 0.3 or 1 µg Ag + SLA. Neutralization activity with 3 µg Ag + SLA was significantly higher than all other groups (p < 0.0001), with 76% neutralization vs. 10% with antigen alone (Fig. 3C). When compared to the antigen alone control, significantly higher levels of neutralization (i.e., 32 to 43%; p < 0.001) were also seen with the serum of mice dosed with 0.3 or 1 µg Ag + SLA. SLA archaeosomes also induced spike-specific cellular responses in an antigen dose-dependent manner (Fig. 3D). A significant increase over antigen alone, was seen in mice immunized with as little as 0.1 µg Ag + SLA, with an average of 6 vs. 22 IFN-γ^+^ spot forming cells (SFCs)/10^6^ splenocytes (p < 0.001). Again, the highest levels of responses were observed with 3 µg Ag + SLA, which induced an average of 303 IFN-γ^+^ SFCs/10^6^ splenocytes, which was significantly higher than what was observed in all other groups (p < 0.0001), except 1 µg Ag + SLA (166 IFN-γ^+^ SFCs/10^6^ splenocytes).

### Comparison of immunogenicity of SmT1 with TLR agonist-based adjuvants or SLA/TLR agonist combinations

**Figure 4:**
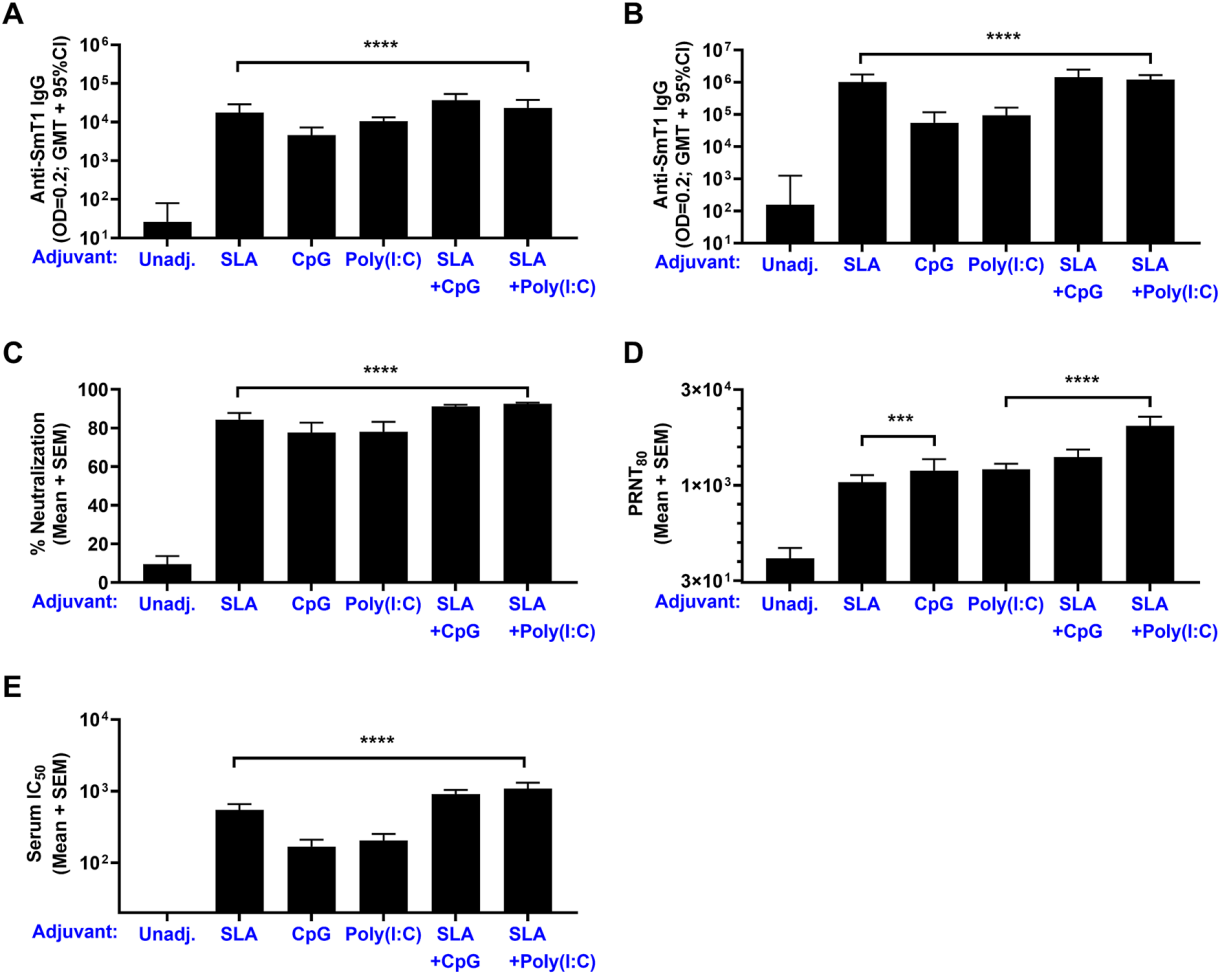
Humoral immune response with SmT1-based vaccine formulations adjuvanted with SLA, CpG and Poly(I:C) alone or in combination. C57BL/6 mice (n = 10/group) were immunized i.m. with 1 µg of SmT1 alone or with SLA, CpG and Poly(I:C) alone or in combination on Days 0 and 21. Serum was collected on Days 20 (**A**) and 28 (**B**) and analyzed by ELISA to determine the antibody titers. Grouped data is presented as geometric mean + 95% confidence interval. Serum from Day 28 was also analyzed at a final serum dilution of 1:75 by cell-based surrogate neutralization assay (**C**). Serial dilutions of serum were also tested in a SARS-CoV-2 plaque reduction neutralization titer assay (**D**) or plate-based spike-ACE2 neutralization assay (**E**). Grouped data is presented as mean + standard error of mean. Statistical significance of differences between the adjuvanted groups vs. the unadjuvanted control group is shown: ***p < 0.001 and ****p < 0.0001 by one-way ANOVA followed by Tukey's multiple comparisons test.

We next sought to evaluate the activity of other adjuvant types (i.e., TLR agonists CpG or Poly(I:C)) when formulated with SmT1. As SLA archaeosomes have been shown to previously synergize with these TLR agonists^40,41^, we also evaluated combination adjuvant formulations containing SLA + CpG or SLA + Poly(I:C). Mice (n = 10 per group) were immunized on Days 0 and 21 with 1 µg of the SmT1 alone or adjuvanted with SLA, CpG, Poly(I:C), SLA + CpG or SLA + Poly(I:C). A suboptimal dose of 1 µg antigen was selected to better allow detection of potential synergy between adjuvants. All adjuvanted formulations induced significantly higher antibody titers than antigen alone following a single vaccine dose (Fig. 4A). Antigen-specific antibody titers were further enhanced following a second vaccine dose, with SmT1 + SLA inducing > tenfold higher titers (GMT of 1,001,596) than formulations adjuvanted with CpG or Poly(I:C) alone (55,244 and 93,114, respectively; Fig. 4B; p < 0. 01). A slight 1.2–1.4-fold increase in antibody titers was seen with SLA + Poly(I:C) and SLA + CpG vs. SLA alone, but this did not reach a level of statistical significance. The activity of the immunized mouse serum was confirmed in the cell-based surrogate neutralization assay, where all adjuvanted formulations induced an average of > 75% neutralization (Fig. 4C). Importantly, the serum of mice immunized with adjuvanted vaccine formulations also significantly reduced the ability of SARS-CoV-2 virus to infect Vero E6 cells and form plaques (Fig. 4D). Serum titers capable of neutralizing the number of plaques by 80% (PRNT_80_) were measured to be > 1000 vs. 73 for all adjuvanted formulations and antigen alone, respectively (p < 0.001). Finally, a plate-based neutralization assay was utilized to further delineate the activity of the vaccine-induce antibodies by determining the concentration of serum necessary to reduce the binding of soluble human ACE2 protein to plate-bound spike by 50% (IC_50_). While all adjuvanted formulations induced superior inhibition to antigen alone (p < 0.0001, Fig. 4E), there were also significant differences between the adjuvanted groups, mirroring the Ab ELISA results seen in Fig. 4B. CpG or Poly(I:C) adjuvanted formulations had average IC_50_ of 167–203, while SmT1 formulated with SLA, SLA + CpG and SLA + Poly(I:C) had significantly higher average IC_50_ (p < 0.01): mean of 549, 907 and 1085, respectively. There was no significant difference between the SLA-containing formulations.

**Figure 5:**
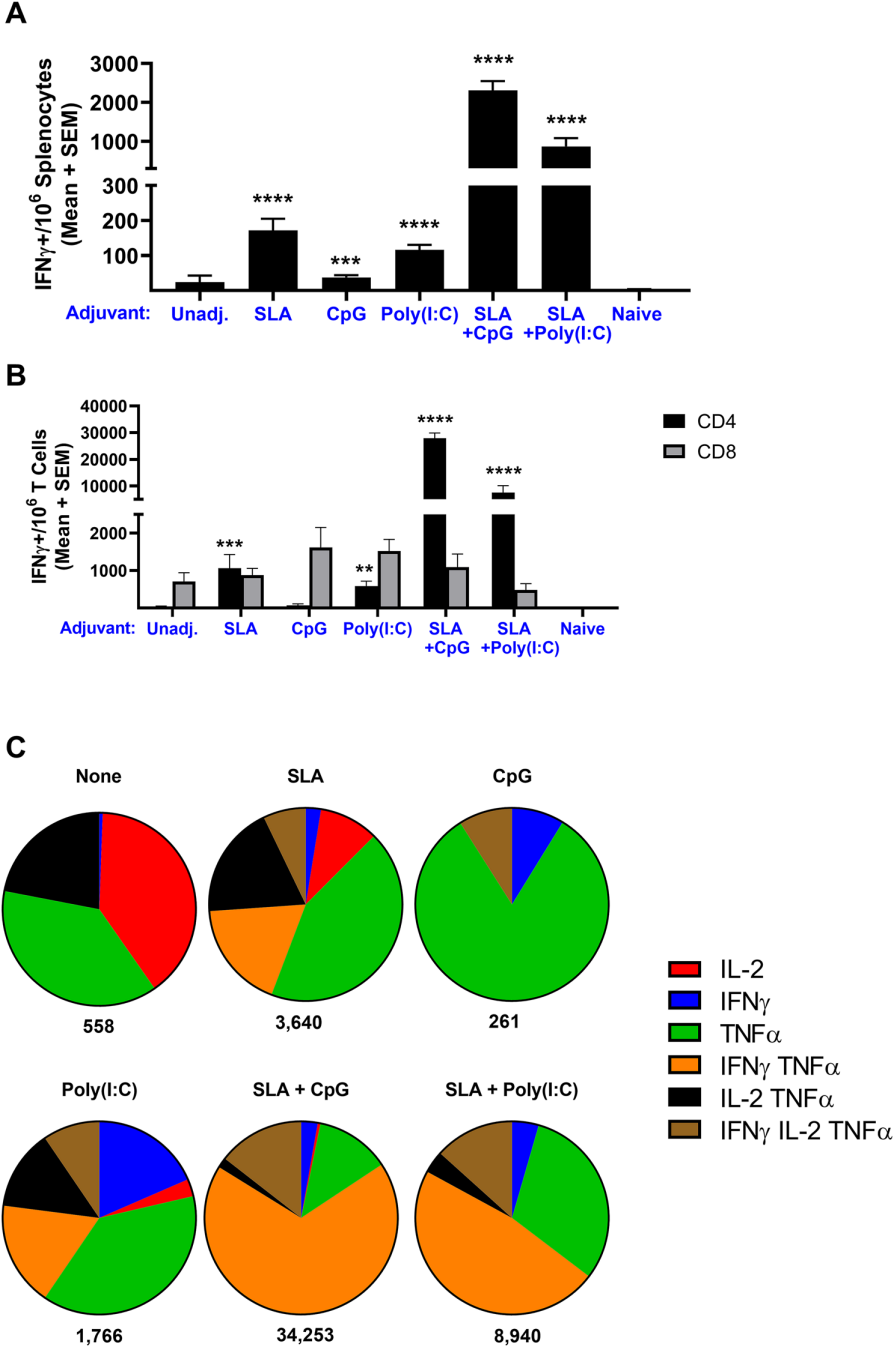
Cellular immune response with SmT1-based vaccine formulations adjuvanted with SLA, CpG and Poly(I:C) alone or in combination. C57BL/6 mice were immunized i.m. with 1 µg of SmT1 alone or with SLA, CpG and Poly(I:C) alone or in combination on Days 0 and 21. Splenocytes were harvested on day 28 and analyzed by IFN-γ^+^ ELISpot (n = 10/group) when stimulated by spike peptide pools or media alone (**A**). Similarly, intracellular cytokine staining (ICCS; n = 5/group) was conducted on the splenocytes to measure levels of IFN-γ^+^ CD4^+^ or IFN-γ^+^ CD8^+^ T cells when stimulated by a spike peptide pools or media alone (**B**). Grouped data is presented as mean + standard error of mean. The frequency of cells expressing IFN-γ, TNF-α, and IL-2 alone or in combination as assessed by ICCS is displayed (median per group) with the total number of cytokine-positive cells per million CD4^+^ T cells indicated below the pie chart (**C**). Values obtained with media alone were subtracted from those measured in the presence of the peptide pool. Statistical significance of differences between the adjuvanted groups vs. the antigen alone control group is shown: **p < 0.01, ***p < 0.001 and ****p < 0.0001 by one-way ANOVA followed by Tukey's multiple comparisons test.

Splenocytes collected on Day 28 were evaluated by IFN-γ ELISpot and intracellular cytokine staining (ICCS) to characterize the antigen-specific cellular responses induced by these vaccine formulations. While all adjuvants induced significantly higher levels of cellular responses as measured by ELISpot (Fig. 5A), the combination adjuvant formulations induced the highest levels with SmT1 adjuvanted with SLA + CpG and SLA + Poly(I:C) inducing an average of 2,308 and 868 IFN-γ^+^ SFCs/10^6^ splenocytes, respectively. The levels in mice immunized with the SLA + CpG-adjuvanted formulation were significantly higher than seen in all other groups (p < 0.05). Amongst the single-adjuvanted formulations, the activity of SLA and Poly(I:C) was significantly higher than CpG (p < 0.05), inducing an average of 172, 117 and 38 IFN-γ^+^ SFCs/10^6^ splenocytes, respectively. By ICCS, we were able to delineate whether the IFN-γ^+^ cells were of the CD4^+^ and CD8^+^ T cell subtype. While IFN-γ^+^ CD8^+^ T cells were seen in all groups, there was no significant difference between the adjuvanted groups and the antigen alone control (Fig. 5B). Conversely, mice treated with some of the adjuvanted formulations had significantly higher levels of IFN-γ^+^ CD4^+^ T cells than those immunized with the antigen alone control. The hierarchy between groups mirrored the ELISpot results with the SLA + CpG-adjuvanted formulation inducing an average of 27,938 IFN-γ^+^ cells/10^6^ CD4^+^ T cells. Depending on the vaccine formulation, a large proportion of the antigen-specific CD4 cells were positive for multiple cytokines (i.e., 9%–83%; Fig. 5C). The percentage of cytokine-positive CD4^+^ T cells expressing more than one cytokine in mice immunized with SLA, Poly(I:C), SLA + CpG and SLA + Poly(I:C)-adjuvanted formulations was 44%, 40%, 83%, and 63%, respectively.

### Immunogenicity and efficacy of SmT1 vaccine formulations in SARS-CoV-2 hamster challenge model

**Figure 6:**
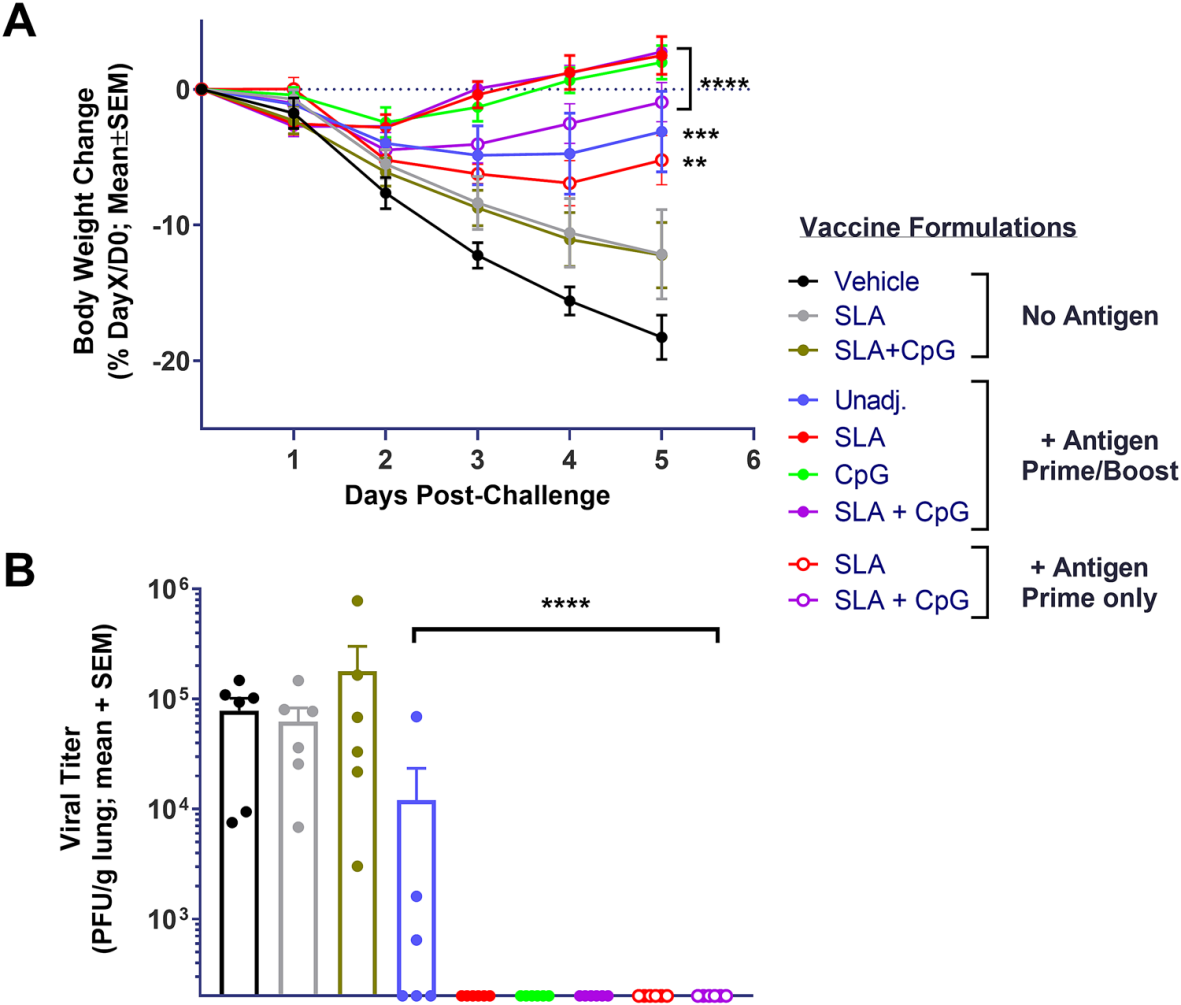
Efficacy of SmT1-based vaccine formulations adjuvanted with SLA, CpG or SLA + CpG in hamster challenge model. Syrian Golden hamsters (n = 6/group) were immunized i.m. with 3 µg of SmT1 alone or with SLA and alone or in combination on Days 0 ± 21. As negative controls, hamsters received either vehicle or adjuvant alone. On Day 35, hamsters were challenged intranasally with 1 × 10^5^ PFU of SARS-CoV-2 (**A**). On Day 5, lungs were collected and viral load measured by plaque assay (**B**). Grouped data is presented as mean + standard error of mean. Statistical significance of differences for the groups vs. the vehicle control group is shown: **p < 0.01, ***p < 0.001 and ****p < 0.0001 by two-way (**A**) or one-way (**B**) ANOVA followed by Tukey's multiple comparisons test.

A hamster challenge experiment was conducted to validate the ability of the most immunogenic vaccine formulations above to mediate protection from SARS-CoV-2 infection. Hamsters (n = 6/group) were immunized on Days 0 (prime) and 21 (boost) with various vaccine formulations: 3 µg of SmT1 alone or adjuvanted with SLA, CpG or SLA + CpG. As negative controls, animals received either vehicle alone, SLA or SLA + CpG without any antigen. The adjuvant alone groups would capture the impact of innate immune activation by the adjuvants on viral challenge. As efficacious single dose vaccine regimens would be highly desirable, separate groups of animals were immunized with 3 µg SmT1 adjuvanted with either SLA or SLA + CpG on Day 0 only. On Day 35, animals were challenged intranasally with SARS-CoV-2 and monitored for body weight loss for 5 days. On Day 40, animals were euthanized and lungs collected for the quantification of viral load by plaque assay. Animals immunized with vehicle continued losing weight during the course of the study with an average ~ 18% reduction in body weight measured by Day 5 post-challenge (Fig. 6A). Animals treated with SLA or SLA + CpG without antigen did have slightly less body weight loss than the vehicle controls, but this was not statistically significant. Immunization with any of the vaccine regimens containing antigen led to a significant decrease in body weight loss. The adjuvanted prime/boost regimens (SmT1 w/SLA, CpG or SLA + CpG) greatly protected the hamsters with their average body weight loss never exceeding 3% and a steady gain in body weight seen thereafter. While not as effective, a significant prevention of body weight loss was also observed in animals receiving the prime only vaccine regimens (SmT1 w/ SLA or SLA + CpG) or prime/boost of antigen alone. The ability of the vaccine formulations to protect from viral infection was confirmed when measuring the viral load in the lungs on Day 5. Average viral loads > 6 × 10^4^ PFU/g lung tissue were seen in hamsters treated with vehicle or adjuvant alone (Fig. 6B). No detectable viral titers were observed in any of the lungs of hamsters immunized with adjuvanted SmT1 formulations (prime only or prime/boost regimens). The antigen alone formulation did lead to a significant decrease in average viral titers, but virus was still detectable in 3/6 animals.

**Figure 7:**
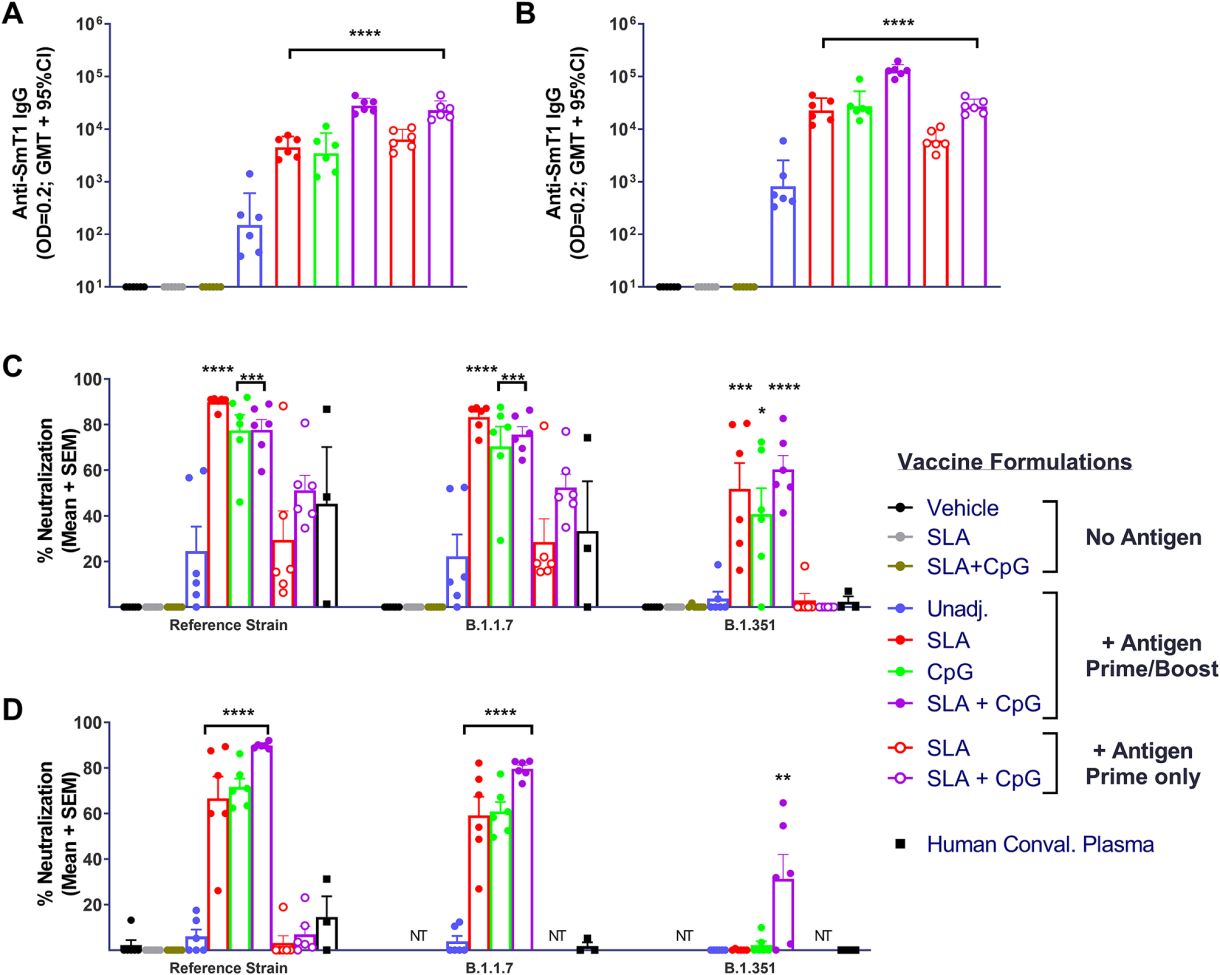
Humoral immune response with SmT1-based vaccine formulations in hamsters. Syrian Golden hamsters (n = 6/group) were immunized i.m. with 3 µg of SmT1 alone or with SLA, CpG or SLA + CpG on Days 0 ± 21. Serum was collected on Days 21 (**A**) and 34 (**B**) and analyzed by ELISA to determine the antibody titers. Grouped data is presented as geometric mean + 95% confidence interval. Serum from Day 34 was also analyzed at a final serum dilution of 1:25 (**C**) and 1:75 (**D**) by cell-based surrogate neutralization assay. Grouped data is presented as mean + standard error of mean. Statistical significance of differences for the adjuvanted groups vs. the antigen alone control group is shown: **p < 0.01, ***p < 0.001 and ****p < 0.0001 by one-way ANOVA followed by Tukey's multiple comparisons test.

Serum was collected from animals prior to vaccination on Days 0 and 21, as well as on Day 34 (1 day prior to viral challenge). No antigen-specific antibodies were detected in any of the groups on Day 0 (data not shown), as well as the control groups immunized with vehicle or adjuvant alone at Days 21 and 34 (Fig. 7A,B). Anti-spike IgG titers were observed in all hamsters immunized with antigen alone following a single vaccine dose, GMT (lower & upper 95% CI) of 150 (37 & 605) (Fig. 7A). The adjuvanted formulations induced significantly higher antigen-specific antibody titers than antigen alone (P < 0.0001). At Day 34, titers increased ~ fivefold in groups that received a boost immunization at Day 21, while they were virtually unchanged in the groups immunized on Day 0 only. Despite some of the groups only receiving a single prime vaccine dose, titers were still significantly higher in all the groups immunized with the adjuvanted formulations than in the antigen alone group (p < 0.0001; Fig. 7B). The prime only vaccine regimens of SmT1 adjuvanted with SLA or SLA + CpG induced GMT of 6,050 and 26,801, respectively. Amongst the groups that received the prime/boost regimens, the SLA + CpG-adjuvanted formulation induced significantly higher titers than the single adjuvant formulations: GMT of 127,989 vs. 22,629–27,430 with SLA or CpG-adjuvanted SmT1 (p < 0.01). The activity of the immunized hamster serum was confirmed in the cell-based neutralization assay (reference strain in Fig. 7C,D), with the ranking between groups similar to that observed above with protection from body weight loss (Fig. 6A). At a serum dilution of 1:25, serum from hamsters receiving the prime only vaccine regimens (SmT1 w/ SLA or SLA + CpG) or the prime/boost of antigen alone demonstrated an average neutralization of 25–51%. Meanwhile, the serum from hamsters receiving the adjuvanted prime/boost regimens had significantly higher neutralization (mean of > 75%) than the antigen alone control (p < 0.001). When assaying a panel of > 20 convalescent samples in this assay we saw mostly weak and variable levels of neutralization (data not shown). Three human plasma controls were selected and assayed side-by-side with the hamster samples: 2 with the strongest activity (NIBSC 20/130 and 20/162) and a negative control (NIBSC 20/B764) with no serological activity to SARS-CoV-2. The negative control yielded 1% neutralization, while 48 or 87% neutralization was detected with the strongly active convalescent samples. When serum was further diluted to 1:75, the serum from animals receiving prime/boost regimen of SmT1 with SLA, CpG or SLA + CpG still had significantly higher neutralization than antigen alone controls (p < 0.0001), while weak neutralization was seen with all other groups and the human controls.

To address the ability of our vaccines to potentially protect against SARS-CoV-2 variants, we adapted our cell-based neutralization assay to evaluate the ability of serum to block binding of B.1.1.7 and B.1.351 spike proteins to Vero E6 cells. Even though the hamsters were immunized with antigen corresponding to the reference SARS-CoV-2, the immunized serum was still capable of strongly blocking the binding of the variant spike to cells. Across the groups, trends with B.1.1.7 were largely similar to those with the reference strain (Fig. 7C,D). Neutralization activity was weaker with B.1.351, with serum from the prime only animals or those immunized with antigen alone no longer showing any activity at either of the serum dilutions tested (similar to human convalescent samples). Importantly, all of the adjuvanted prime/boost vaccine regimens induced sufficiently strong antibody responses to significantly neutralize binding of B.1.351 spike when tested at the 1:25 dilution (Fig. 7C). Even at the more stringent 1:75 dilution, serum from hamsters receiving the SmT1 with SLA + CpG had significantly higher neutralization capacity of B.1.351 spike than the antigen alone controls (p < 0.01).

## Discussion

In this study, we have brought together three proprietary technologies; a resistin-trimerized spike antigen, our CHO2353™ cell manufacturing platform, and SLA archaeosome adjuvant, to formulate and evaluate a novel SARS-COV2 vaccine candidate. We have previously demonstrated our ability to produce high yields of a novel spike antigen, SmT1, from CHO2353™ cells^30,42^. To mimic the structure of the native spike glycoprotein, a human resistin domain was fused to the spike ectodomain allowing the proteins to form highly pure trimeric structures. Herein, we demonstrate the strong immunogenicity and efficacy of adjuvanted vaccine formulations based on SmT1. Additionally, the generated antibodies were capable of cross-neutralizing binding of spike protein from multiple variants of concern. The ability of our CHO2353™ cell manufacturing platform to generate high yields of antigen combined with the strong adjuvanticity and dose sparing effect of SLA archaeosomes indicates the approach outlined in this study could support rapid and cost-effective large-scale vaccination efforts.

Multiple SARS-CoV-2 vaccine formulations have been evaluated in preclinical and clinical studies^3–9,19,21,26,43,44^. Some of the most advanced protein subunit vaccine formulations include Novavax’s NVX-CoV2373 adjuvanted with Matrix-M and Clover Biopharmaceutical’s S-Trimer adjuvanted with Alum/CpG. Both formulations demonstrated immunogenicity and efficacy in preclinical models^21,45^, while demonstrating immunogenicity in human clinical trials^19,46,47^. NVX-CoV2373’s spike antigen is produced via the baculovirus/sf9 insect cell system, and relies on the native transmembrane domain for the formation of homotrimers. The requirement for solubilisation of membrane-incorporated spike during purification of this antigen would likely complicate downstream purification when compared to soluble spike constructs such as SmT1. To improve stability, they have included similar mutations as our SmT1 above, namely the abolition of the furin S1/S2 cleavage site and introduction of stabilizing prolines in the S2 domain. Clover’s S-Trimer antigen is produced in Chinese hamster ovary (CHO) cells and includes a human procollagen trimerization domain in place of the native transmembrane domain. However, it does not include the stabilizing mutations listed above, leading to the detection of small fractions of cleaved product (i.e. S1) in their protein preparations^21^. Without a head-to-head comparison, it is difficult to directly compare the immunogenicity of our vaccine formulation to those mentioned above. It is encouraging that we did obtain similar immunogenic profiles (strong neutralizing Ab activity, antigen-specific cellular immune responses and protection in a challenge model) when immunizing with comparable levels of antigen. In addition, these formulations do demonstrate the ability of antigens with differing properties to induce efficacious antigen-specific immune responses when paired with an appropriate adjuvant. SmT1’s inclusion of stabilizing mutations and strong trimerization domains along with our CHO2353™ platform allows us to generate a highly immunogenic and pure antigen at high yields without any cleaved fractions. The importance of these features may be virus variant dependant. While we have been able to generate stable and equally immunogenic antigen formulations based on the reference SARS-CoV-2 without the stabilizing prolines, these residues appear to be required for the optimal manufacturability of SmT1 (B.1.351) in our platform (unpublished data).

With regards to safety, no major concerns have been identified so far with protein subunit-based SARS-CoV-2 vaccines in preclinical or clinical studies. While rare side effects can only be identified when a large amount of people are vaccinated (either in phase 3 clinical trials or post-approval), protein subunit vaccines have a long track record of safety. Some of the observed side effects with the mRNA and viral vector vaccines appear to be platform specific. For example, both the AstraZeneca and Janssen adenoviral vaccines showed low frequency of clotting disorders, while both the Moderna and Pfizer-BioNtech vaccines showed low frequency of myocarditis and severe anaphylaxis^14–16^. The induction of thrombocytopenia by adenoviral gene transfer vectors has been reported previously, with the vectors shown to directly bind and activate platelets^48^. With protein subunit vaccines, safety will depend on both the adjuvant and antigen profiles; certainly, the degree of purification possible for subunit vaccines should be superior to adenoviral vector-based vaccines, which have been shown to include substantial levels of host cell-derived proteins^49^.

With SmT1, use of resistin should focus responses on the intended viral domains when administered to humans. While there is a small risk of inducing antibodies to human resistin, breaking tolerance is generally quite difficult to achieve. Theoretically when administered to humans, the antigen-specific immune response should focus on the foreign epitopes, with responses to the human resistin domain being non-existent or weak. To our knowledge, auto-immune responses to resistin have not been reported. In fact, antibody strategies targeting resistin have been employed preclinically as potential treatments for obesity/diabetes with no safety concerns identified^50^. Future studies evaluating the safety and immunogenicity of resistin-based antigens in a self-antigen setting will be important.

While multiple vaccines are based on the entire spike ectodomain, approaches targeting subdomains such as the RBD could prove effective. This approach would focus on the immune response on the critical domain mediating cellular entry/infection. In our hands, immunization with NTD, RBD, S1 or S2 did not yield high levels of neutralizing antibodies. While this may be expected for the domains that do not include RBD, the lack of strong responses with the RBD and S1 antigens may be due to their overall structure and size. Not only is the more immunogenic SmT1 format longer in sequence, but is able to form larger trimeric complexes. Antigen size can affect its ability to drain to lymph nodes and interact with the immune compartment^51^. A number of strategies utilizing RBD-based vaccine formulations have been shown to be immunogenic/efficacious in preclinical models^52–54^. Interestingly, some of these approaches rely either on carriers (i.e. capsid-like particles, nanoparticles) or T helper epitopes (i.e. tetanus toxoid peptide) to enhance the immunogenicity of RBD. While these strategies are effective in inducing neutralizing antibodies, they do limit the number of available T cell epitopes. Of all antigens tested in our mouse model, the NTD-based formulation appeared to stimulate the strongest responses by IFN-γ ELISpot. This is likely due to NTD containing the epitope best recognized in our mouse model and to the administration of an increased number of epitope molecules in our study with NTD, as all vaccine formulations contained the same amount of antigen by weight. A larger protein with a potentially larger repertoire of epitopes would be more likely to induce T cell responses in a heterogeneous human population with differing MHC haplotypes. Vaccine-induced T cells could be an important strategy to contain existing and future variants of concern, as their reactivity has been shown to not be impacted by the mutations^22^. As mutations accumulate in the variants, they will be less able to circumvent the immune response if multiple epitopes covering different portions of the protein are targeted simultaneously by the humoral and cellular branches of the immune response. Strategies based on RBD could circumvent potential antibody-dependant enhancement (ADE) of disease caused by non-neutralizing Ab as seen with preclinical vaccines against the original SARS-CoV^55^. This may not be relevant to SARS-CoV-2, as numerous vaccine strategies based on the entire spike ectodomain have yet to yield any evidence of ADE^56^.

With a protein subunit vaccine, the magnitude and profile (humoral and/or cellular) is also dependent on the adjuvant used. SLA archaeosomes constitute a well-tolerated robust adjuvant system based on a single archaea-derived lipid that induces strong humoral and cellular immune responses to a variety of antigens when simply admixed^34,36–38^. In addition, it has been shown to synergize with other adjuvant types, namely Poly(I:C) or CpG^40,41^. While the exact mechanism behind this activity has yet to be elucidated, it is interesting to note that when a panel of adjuvants were screened for synergy with SLA, nucleic-acid based agonists of intracellular TLRs generated the strongest enhancement of antigen-specific immune responses. This could be due to a number of factors such as their chemical nature, cellular localization and/or ability to stimulate distinct immune activation pathways. This adjuvant activity was further confirmed with SmT1, which was also compatible with other adjuvant types including aluminum salts and TLR agonists. We did see differences in adjuvant activity in our models, but they were sometimes assay and species dependant. A single dose of SmT1 adjuvanted with SLA or SLA/CpG induced protective immune responses in our viral challenge model and completely abolished detectable viral load in the lungs at Day 5 post infection. In addition, vaccinated serum was better able than convalescent human serum to neutralize binding of spike from the reference strain or variants of concern. Some of the mutations in B.1.1.7 and B.1.351 do fall in the peptide array hotspots identified with our vaccine-induced antibodies^57,58^. Immunized hamster serum mediated similar levels of neutralization against spike protein from the reference and B.1.1.7 strains, as was shown by others^59^. In the case of B.1.351, we did detect a reduced capacity for neutralization by our immunized hamster serum, especially in animals receiving a single vaccine dose. Again, this was similar to previous reports showing reduced neutralization of B.1.351 by antibodies generated against antigen from the reference strain (via infection or vaccination)^23,24^. Interestingly, B.1.351 neutralization was still strongly mediated by the serum of animals receiving a double dose adjuvanted vaccine regimen, especially the formulation adjuvanted with SLA + CpG. As vaccine regimens with similar levels of neutralization against the reference strain were protective in our challenge model, properly adjuvanted vaccine formulations including the reference-based SmT1 antigen could cross-protect against challenge with these variants. As the virulence of the variants may differ, it will be important to address this in future challenge studies. In addition, the compatibility of our vaccine platform with spike proteins based on the variants of concern will be confirmed through immunogenicity/efficacy studies.

In summary, our data demonstrate that resistin-trimerized SmT1 is a novel robust antigen format. Furthermore, combining it with an adjuvant such as SLA yields robust humoral and cellular immune responses targeting SARS-CoV-2 spike protein. SmT1 is also easily amenable to be used with other adjuvants such as CpG or Alum. Due to the importance of an accelerated vaccine rollout to mitigate the spread of variants of concern^60^, the limited availability of vaccines/adjuvants and certain drawbacks associated with current vaccines, our proprietary technologies of rapid antigen production (CHO2353™) as well as archaeosome adjuvants provide a promising candidate for next generation SARS-CoV-2 vaccines. As SLA-adjuvanted formulations have yet to be tested in humans, it is possible that its activity/safety profile will not mirror what has been observed in preclinical models. We are currently conducting toxicology studies that would enable us to advance these promising vaccine formulations, so that their safety/efficacy can be evaluated in a clinical setting. As the COVID-19 pandemic evolves and we get a better understanding of the longevity and cross-protection of the immune responses generated by the various clinical vaccines, we will have to assess how best to evaluate and deploy novel vaccine formulations.

## Materials and methods

### Animals and virus

Female C57BL/6 mice (6–8 weeks old) and Syrian golden hamsters (81–90 g) were obtained from Charles River Laboratories (Saint-Constant, Canada). Animals were maintained at the small animal facility of the National Research Council Canada (NRC) in accordance with the guidelines of the Canadian Council on Animal Care. All procedures performed on animals in this study were approved by our Institutional Review Board (NRC Human Health Therapeutics Animal Care Committee) and covered under animal use protocols 2020.06 & 2020.10. All experiments were carried out in accordance with the ARRIVE guidelines.

SARS-CoV-2 isolate Canada/ON/VIDO-01/2020 (obtained from the National Microbiology Lab, Winnipeg, Canada) was propagated and quantified on Vero E6 cells. Whole viral genome sequencing was carried out to confirm exact genetic identity to original isolate. Passage 3 virus stocks were used in all subsequent experiments.

### Recombinant antigens and proteins

SmT1 was designed as previously described^30^. Briefly, SARS-CoV-2 sequences (amino acids 1–1208 derived from Genbank accession number MN908947) were codon-optimized for Chinese Hamster Ovary (CHO) cells and synthesized by GenScript. Within the construct, the spike glycoprotein was preceded by its natural N-terminal signal peptide and fused at the C-terminus to the human resistin (accession number NP_001180303.1, amino acids 23–108), followed by FLAG and 6xHis Tags. Mutations were added to stabilize the generated spike protein as previously described; amino acids 682–685 (RRAR) and 986–987 (KV) were replaced with GGAS and PP, respectively^32,61^. Constructs were then cloned into pTT241 plasmid, expressed from stable inducible CHO^2353^™ pools and cell supernatants collected 8–10 days following cumate induction^42^. Proteins were purified with immobilized metal affinity column with Nickel Sepharose excel resin (GE Healthcare, Little Chalfont, UK). The SmT1 purified Reference Material can be obtained at the NRC webstore^62^. The N-terminal domain (NTD), Receptor binding domain (RBD), S1 and S2 proteins corresponding to amino acids 1–307, 331–521, 16–686 and 687–1208 of the spike glycoprotein, respectively, were generated by CHO^BRI/55E1^ transfection as described previously^30^. The natural signal peptide for SARS-CoV-2 spike (a.a. 1–15) found within the NTD was used for S2 as well, while a signal peptide derived from human IL-10 was utilized for the RBD and S1 constructs. Antigens were tested with Endosafe® cartridge-based Limulus amebocyte lysate test (Charles River Laboratories, Charleston, SC, USA) to confirm lack of endotoxin contamination.

The human ACE2 (UniProtKB-Q9BYF1) cDNA was codon-optimized for expression in CHO cells and synthesized by GenScript. The construct encodes an N-terminal human interleukin-10 signal peptide (MHSSALLCCLVLLTGVRA) followed by a (Twin-)Strep-tag II—(His)_6_—FLAG tag fused to the N-terminus of the mature ACE2 receptor ectodomain (amino acids 20–613: TIEE…WSPY). A biotin acceptor peptide sequence (BAP: GLNDIFEAQKIEWHE) was added in-frame at the C-terminus of ACE2. The cDNA was cloned into pTT5® expression plasmid using EcoRI–BamHI restriction sites. The hACE2-BAP cDNA was expressed by transient transfection of CHO^BRI/55E1^ cells as described above with the addition of 5% (w:w) of pTT5-BirA (*E. coli* biotin ligase) expression plasmid as described previously^63^. Clarified culture supernatant harvested at 8 days post-transfection was purified by IMAC on Nickel Sepharose excel as described above. IMAC eluate was subjected to a second affinity purification step using Strep-Tactin XT Superflow (IBA, Gottingen, Germany), following manufacturer’s instructions. Strep-Tactin eluate was buffer-exchanged into DPBS, aliquoted and stored at − 80 °C.

### Immunization and sample collection

Mouse experiments (n = 8–10 per group) consisted of 2 separate equal sized cohorts, where animals were treated identically but had procedures conducted on different days. Data from both cohorts was combined and included for analysis. Antigen and adjuvant vaccine components were admixed and diluted in phosphate buffered saline (PBS; Thermo Fisher Scientific, Waltham, MA, USA) prior to administration in a final volume of 50 µL per dose. Sulfated lactosyl archaeol (SLA) archaeosomes are proprietary NRC adjuvants that were prepared as previously described^64^. Levels of endotoxin in the SLA archaeosomes were verified by the Endosafe® cartridge-based Limulus amebocyte lysate test (Charles River Laboratories) and confirmed to be < 0.1 EU per mg. AdjuPhos®, CpG (ODN 1826) and Poly(I:C) (all from Invivogen, San Diego, CA, USA) were prepared as per manufacturer’s instructions. Adjuvant dose levels were based on data from previous studies with 1 mg SLA, 20 µg CpG, 50 µg Poly(I:C) and 50 µg Al^3+^ included per dose.

Animals were immunized by intramuscular (i.m.) injection (50 µL) into the left tibialis anterior (T.A.) muscle on Days 0 and/or 21 with various vaccine formulations as described above. On Day 28, mice were anesthetized with isoflurane and then euthanized by cervical dislocation prior to collection of spleens for measurement of cellular immune responses by IFN-γ ELISpot and/or intracellular cytokine staining. Mice were bled via the submandibular vein on Days 20 and 28 with recovered serum used for quantification of antigen specific IgG antibody levels and various neutralization assays. Hamsters were bled via the anterior vena cava on Days 0, 21 and 34 with recovered serum analyzed by similar serological assays.

On Day 35 hamsters were challenged intranasally by 1 × 10^5^ PFU of SARS-CoV-2. Animals were monitored daily for body weight change and clinical signs (lethargy, grimace, ruffling). On Day 40, animals were euthanized by exposure to CO_2_ (following anesthesia by isoflurane) and tissues collected for assessment of viral load by plaque assay as described below.

### Anti-spike ELISA

Anti-spike total IgG titers in serum were quantified by ELISA. Briefly, 96–well high-binding ELISA plates (Thermo Fisher Scientific) were coated overnight at room temperature (RT) with 100 µL of 0.3 µg/mL SmT1 protein (same as used for immunization) diluted in PBS. Plates were washed five times with PBS/0.05% Tween20 (PBS-T; Sigma-Aldrich, St. Louis, MO, USA), and then blocked for 1 h at 37 °C with 200 µL 10% fetal bovine serum (FBS; Thermo Fisher Scientific) in PBS. After the plates were washed five times with PBS-T, 3.162-fold serially diluted samples in PBS-T with 10% FBS were added in 100 µL volumes and incubated for 1 h at 37 °C. After five washes with PBS-T (Sigma-Aldrich), 100 µL of goat anti-mouse IgG -HRP (1:4,000, Southern Biotech, Birmingham, AL, USA) or goat anti-hamster IgG-HRP (1:32,000, Southern Biotech) was added for 1 h at 37 °C. After five washes with PBS-T, 100 µL/well of the substrate o-phenylenediamine dihydrochloride (OPD, Sigma-Aldrich) diluted in 0.05 M citrate buffer (pH 5.0) was added. Plates were developed for 30 min at RT in the dark. The reaction was stopped with 50 µL/well of 4 N H_2_SO_4_. Bound IgG Abs were detected spectrophotometrically at 450 nm. Titers for IgG in serum were defined as the dilution that resulted in an absorbance value (OD 450) of 0.2 and were calculated using XLfit software (ID Business Solutions, Guildford, UK). Samples that does not reach the target OD were assigned the value of the lowest tested dilution (i.e. 10) for analysis purposes. No detectable titers were measured in serum samples from naïve control animals.

### Peptide microarray assay

The replitope assay was performed using a commercially available microarray and associated kit (JPT Peptide Technologies GmbH, Berlin, Germany) according to manufacturer’s instructions. The microarray displays 315 peptides as 15mers with 11 amino acid overlap of the SARS-CoV-2 spike Glycoprotein (Swiss-Prot ID: P0DTC2—UniProt release 2020_02). Briefly, sera from immunized mice was diluted 1:200 in provided blocking buffer with 150µL of sample added to the microarray in triplicate. The microarray was covered and incubated on an orbital shaker at 300 rpm and 30 °C for 1 h. The microarray was washed 4 times with 300 µL of Tris Buffered Saline-0.1% Tween20 (TBS-T from JPT Peptide Technologies). The secondary antibody, consisting of goat anti-mouse IgG DyLight 650 (Thermo Fisher Scientific), was diluted to 1 µg/mL and added at 150 µL per well. The covered plate was once again incubated on an orbital shaker at 300 rpm and 30 °C for 1 h. As before, the microarray was washed 4 times with 300µL of TBS-T followed by 2 washes with 300µL of sterile MilliQ H_2_O. Slides were dried by brief centrifugation prior to analysis on a Genepix 4000A (Molecular Devices, Sunnyvale, CA, USA).

### Vero E6 cell-based surrogate SARS-CoV-2 neutralization assay

This surrogate neutralization assay was performed similarly to conventional pseudoparticle neutralization assays^65^. Briefly, the ability of labeled SARS-CoV-2 spike trimers (SmT1) to bind the surface of Vero E6 cells following co-incubation with sera/plasma was measured. Vero E6 cells were maintained in RPMI 1640 supplemented with 10% FBS, 1% penicillin/streptomycin, 20 mM HEPES, 1 × non-essential amino acids, 1 × Glutamax, 50 µM 2-mercaptoethanol (all from Thermo Fisher Scientific) at 37 °C with 5% CO2. Soluble SmT1, was biotinylated and isolated from free biotin using EZ-Link™ NHS-LC-LC-Biotin (Thermo Fisher Scientific) according to manufacturer’s instructions. Indicated dilutions of mouse/hamster serum or convalescent human plasma (20/130, 20/162, 20/B764 from NIBSC, South Mimms, UK) were mixed with 250 ng of biotinylated spike and 1 × 10^5^ Vero E6 cells (ATCC® CRL-1586™) in the presence of 0.05% azide within a 96-well V-bottom plate (Nunc™, Thermo Fisher Scientific) and incubated for 1 h at 4 °C, while protected from light. Regardless of serum concentration, the final volume of all samples was normalized to 150 µL. Cells were washed with PBS + 1% bovine serum albumin (BSA) + 0.05% Azide and incubated with Streptavidin–phycoerythrin conjugate for 1 h at 4 °C (Thermo Fisher Scientific). After another wash, the cells were fixed using CytoFix™ (Becton Dickinson, Franklin Lakes, NJ, USA) and resuspended in wash buffer + 5 mM EDTA for acquisition on an LSR Fortessa (Becton Dickinson). Spike binding to cells was determined by calculating the Geometric Mean Fluorescence Intensity (MFI) of PE (on singlet cell population) and subtracting the same parameter measured from control cells (incubated in absence of plasma/serum), both above background noise as determined by the negative control, using FlowJo analysis software. Percent neutralization was calculated as follows: % neutralization = 100 – (100 × (Geometric MFI for PE of test sample – Geometric MFI for PE of negative control sample (i.e. cells incubated only with Streptavidin-PE and without spike protein)/(Geometric MFI for PE of positive control sample (i.e., cells incubated with spike without serum/plasma) – Geometric MFI for PE of negative control sample)). For analysis purposes, samples with calculated values ≤ 0 were assigned a value of 0.

### Plate-based surrogate SARS-CoV-2 neutralization assay

The plate-based surrogate neutralization assay measured the ability of immunized serum to block the interaction between a plate-bound spike protein and a soluble human ACE2 receptor protein. Briefly, 96–well high-binding ELISA plates (Thermo Fisher Scientific) were coated overnight at 4 °C with 100 µL of 3 µg/mL SmT1 protein (same as used for immunization) diluted in PBS. Plates were washed five times with PBS-T (Sigma-Aldrich) before each of the following steps until the addition of substrate. Plates were first blocked for 2 h at 37 °C with 200 µL 3% skim milk (Becton Dickinson) in PBS. Serum was 3.162-fold serially diluted in diluent buffer (PBS-T + 0.5% skim milk) with 100 µL of diluted serum added to the plates, followed by incubation for 1 h at 37 °C. Afterwards, 100 µL of hACE2-BAP (50 ng/well) in diluent buffer was added and plates incubated at 37 °C for 1 h. 100 µL of strepatavidin peroxidase polymer (67 ng/well; Sigma-Aldrich) in diluent buffer was then added and plates incubated at 37 °C for 45 min. After the last wash, 100 µL/well of the SureBlue 3,3′,5,5′-Tetramethylbenzidine (TMB) substrate (SeraCare Life Sciences, Milford, MA, USA) was added. Plates were developed for 8 min at RT. The reaction was stopped with 100 µL/well of TMB stop solution (SeraCare Life Sciences). The amount of bound ACE2 was detected spectrophotometrically at 450 nm. To calculate the percent inhibition per well, values were normalized to those obtained in wells incubated with BAP but no serum (0%) and those without both BAP/serum (100%). The dilution of serum mediating 50% inhibition of ACE2 binding per sample was determined using XLfit software (ID Business Solutions).

### ELISpot

The levels of spike glycoprotein specific T cells were quantified by ELISpot using a mouse IFN-γ kit (Mabtech Inc., Cincinnati, OH, USA). Spleens were mechanically minced with the frosted ends of two glass slides and splenocytes were isolated in RPMI media (Thermo Fisher Scientific) containing 10% FBS (Thermo Fisher Scientific), 1% penicillin/streptomycin (Thermo Fisher Scientific), 1% glutamine (Thermo Fisher Scientific) and 55 µM 2-Mercaptoethanol (Thermo Fisher Scientific). Cells were passed through a 70 µm cell strainer and cell yields determined on a Cellometer (Nexcelom, Lawrence, MA, USA). A spike peptide library (JPT Peptide Technologies GmbH) consisting of 315 peptides (15mers overlapping by 11 amino acids with last peptide consisting of a 17mer) was used to stimulate the cells. The library was split into 2 subpools covering either the N-terminal (158 peptides) or C-terminal (157 peptides) half of the spike protein and used to separately stimulate 4 × 10^5^ cells in duplicate at a final concentration of 2 µg/mL per peptide. Final volume per well was 0.2 mL. Cells were also incubated without any stimulants to measure background responses. Plates were incubated for ~ 20 h at 37 °C with 5% CO_2_, at which point the plates were washed and developed according to the manufacturer’s instructions. AEC substrate (Becton Dickenson) was used to visualize the spots. Spots were counted using an automated ELISpot plate reader (Cellular Technology LTD, Beachwood, OH, USA). For each animal, values obtained with media alone were subtracted from those obtained with each of the spike peptide pools, and then combined to yield an overall number of antigen-specific IFN-γ^+^ SFC/10^6^ splenocytes per animal.

### Intracellular cytokine staining

The phenotype (CD4 vs. CD8) and polyfunctionality (expression of IFN-γ, TNF-α, and/or IL-2) of spike-specific T cells were determined by intracellular cytokine staining of splenocytes. Cells (2 × 10^6^ per sample) were stimulated separately with the Spike Glycoprotein peptide subpools as described above in the presence of Golgiplug™ (Becton Dickinson) for ~ 20 h at 37 °C with 5% CO_2_. Cells were also incubated without any peptides to measure background responses. Following incubation, splenocytes were washed with PBS (Thermo Fisher Scientific) and stained with the fixable blue dead cell stain (Thermo Fisher Scientific). Cells were then stained with an antibody cocktail to identify immune cell types through binding of cell surface markers: anti-CD14-BV510 (Becton Dickinson), anti-CD16-BV510 (Becton Dickinson), anti-CD19-BV510 (Becton Dickinson), anti-CD4-APC-Cy7 (Becton Dickinson), and anti-CD8-PerCp-Cy5.5 (Becton Dickinson) diluted in staining buffer (PBS + 2% FBS; Thermo Fisher Scientific). Cells were then washed in staining buffer and permeabilized for intracellular staining using the BD Cytofix/Cytoperm™ kit (Becton Dickinson) according to the manufacturer’s instructions. Samples were then stained with an antibody cocktail to anti-CD3-AF700 (eBioscience, San Diego, CA, USA), anti-CD69-PE-CF594 (Becton Dickinson), anti-IFN-γ-AF488 (Becton Dickinson), anti-TNF-α-BV421 (Becton Dickinson), anti-IL-2-APC (Becton Dickinson), and Granzyme B-PE-Cy7 (eBioscience) diluted in permeabilization wash buffer (Becton Dickinson). All samples were washed and resuspended in staining buffer for acquisition with a BD Fortessa flow cytometer (Becton Dickinson). Cell populations were characterized as follows: Non-T cells and dead cells were excluded based on staining for BV510 and the fixable dye, respectively. Activated CD3 + CD4 + or CD3 + CD8 + T cells were identified through positive staining of the CD69 activation marker prior to classifying them as IFN- γ, TNF-α, and/or IL-2 positive cells. As above with ELISpot, values were background subtracted and then combined for each animal.

### Plaque assay

This assay was performed exclusively within a containment level 3 facility (CL3). Whole left lung from each hamster was homogenized in 1 mL PBS. The samples were centrifuged and the clarified supernatants were used in a plaque assay. The plaque assay, in brief, was carried out by diluting the clarified lung homogenate in a 1 in 10 serial dilution in infection media (1 × DMEM, high glucose media supplemented with 1 × non-essential amino acid, 100 U/mL penicillin–streptomycin, 1 mM sodium pyruvate, and 0.1% bovine serum albumin). Vero E6 cells were infected for 1 h at 37 °C before the inoculum was removed and overlay media was added, which consisted of infection media with 0.6% ultrapure, low-melting point agarose. The cells were incubated at 37 °C/5% CO_2_ for 72 h. After incubation, cells were fixed with 10% formaldehyde and stained with crystal violet. Plaques were enumerated and PFU was determined per gram of lung tissue.

### Plaque reduction neutralization tests (PRNT)

All steps carried out for the PRNT assay was performed in a CL3 facility. Serum samples were inactivated at 56 °C for 30 min and stored on ice. A 1-in-2 serial dilution was carried out using inactivated serum. Diluted serum was incubated with equal volume containing 100 PFU of SARS-CoV-2 at 37 °C for 1 h, followed by infection of Vero E6 cells. Adsorption of virus were carried out for 1 h at 37 °C. Inoculum was removed after adsorption and overlay media as described above was added over the infected cells. The assay was incubated at 37 °C/5% CO_2_ for 72 h. After incubation, cells were fixed with 10% formaldehyde and stained with crystal violet. Controls included naïve animal serum, as well as a no serum, virus-only back-titer control. PRNT_80_ is defined as the highest dilution of serum that results in 80% reduction of plaque-forming units. Samples that does not result in an 80% reduction in PFUs were assigned the value of the lowest tested dilution (i.e. 40) for analysis purposes.

### Statistical analysis

Data were analyzed using GraphPad Prism® version 8 (GraphPad Software). Statistical significance of the difference between groups was calculated by one-way or two-way analysis of variance (ANOVA) followed by post-hoc analysis using Tukey's (comparison across all groups) multiple comparison test. Data was log transformed (except for % neutralization and % body weight loss) prior to statistical analysis. For all analyses, differences were considered to be not significant with p > 0.05. Significance was indicated in the graphs as follows: *p < 0.05, **p < 0.01, ***p < 0.001 and ****: p < 0.0001.

### Institutional review board statement

Mice were maintained at the small animal facility of the National Research Council (NRC) Canada in accordance with the guidelines of the Canadian Council on Animal Care. All procedures performed on animals in this study were approved by our Institutional Review Board (NRC Human Health Therapeutics Animal Care Committee) and covered under animal use protocols 2020.06 & 2020.10. All experiments were carried out in accordance with the ARRIVE guidelines.

## Data Availability

The data presented in this study are available on request from the corresponding author. The data are not publicly available due to privacy concerns.
